# Epigenetic Silencing of Plasmodium falciparum Genes Linked to Erythrocyte Invasion

**DOI:** 10.1371/journal.ppat.0030107

**Published:** 2007-08-03

**Authors:** Alfred Cortés, Celine Carret, Osamu Kaneko, Brian Y. S. Yim Lim, Alasdair Ivens, Anthony A Holder

**Affiliations:** 1 Division of Parasitology, Medical Research Council National Institute for Medical Research (NIMR), London, United Kingdom; 2 Institució Catalana de Recerca i Estudis Avançats (ICREA) and Institute for Research in Biomedicine (IRB), Barcelona, Catalonia, Spain; 3 Pathogen Microarrays Group, The Wellcome Trust Sanger Institute, Cambridge, United Kingdom; 4 Department of Molecular Parasitology, Ehime University Graduate School of Medicine, Ehime, Japan; Seattle Biomedical Research Institute, United States of America

## Abstract

The process of erythrocyte invasion by merozoites of Plasmodium falciparum involves multiple steps, including the formation of a moving junction between parasite and host cell, and it is characterised by the redundancy of many of the receptor–ligand interactions involved. Several parasite proteins that interact with erythrocyte receptors or participate in other steps of invasion are encoded by small subtelomerically located gene families of four to seven members. We report here that members of the *eba, rhoph1/clag, acbp,* and *pfRh* multigene families exist in either an active or a silenced state. In the case of two members of the *rhoph1/clag* family, *clag3.1* and *clag3.2,* expression was mutually exclusive. Silencing was clonally transmitted and occurred in the absence of detectable DNA alterations, suggesting that it is epigenetic. This was demonstrated for *eba-140*. Our data demonstrate that variant or mutually exclusive expression and epigenetic silencing in *Plasmodium* are not unique to genes such as *var*, which encode proteins that are exported to the surface of the erythrocyte, but also occur for genes involved in host cell invasion. Clonal variant expression of invasion-related ligands increases the flexibility of the parasite to adapt to its human host.

## Author Summary

Plasmodium falciparum is responsible for the most severe forms of human malaria. Invasion of host erythrocytes is an essential step of the complex life cycle of this parasite. There is redundancy in many of the interactions involved in this process, such that the parasite can use different sets of receptor–ligand interactions to invade. Here, we demonstrate that the parasite can turn off the expression of some of the proteins that mediate invasion of erythrocytes. Expression can be turned off without alterations in the genetic information of the parasite by using a mechanism known as epigenetic silencing. This is far more flexible than genetic changes, and permits fast, reversible adaptation. Turning on or off the expression of these proteins did not affect the capacity of the parasite to invade normal or modified red cells, which suggests that the variant expression of these genes may be used by the parasite to escape immune responses from the host. Parasite proteins that participate in erythrocyte invasion are important vaccine candidates. Determining which proteins can be turned off is important because vaccines based on single antigens of the parasite that can be turned off without affecting its growth would have little chance of inducing protective immunity.

## Introduction

Invasion of human erythrocytes by merozoites of the malaria parasite P. falciparum is an essential step of the asexual blood cycle of the parasite, which is responsible for all the pathology associated with the disease. Erythrocyte invasion by merozoites of P. falciparum is relatively well characterised at the ultrastructural level [1], but the precise molecular interactions and the role of the specific parasite proteins are still poorly described. Proteins located on the surface of the merozoite probably mediate the initial, reversible contact through low affinity interactions. The next step involves reorientation of the merozoite, such that its apical end, which contains specialised organelles like rhoptries and micronemes, faces the erythrocyte. This is followed by the irreversible formation of a tight moving junction based on high affinity interactions (reviewed in [2]). The micronemal protein EBA-175 is believed to participate in junction formation by interacting with the erythrocyte surface protein glycophorin A [3], but other proteins located in the apical organelles of the merozoite are also likely to be involved. Strong candidates are the proteins encoded by the small gene families *eba* (also known as *dbl-ebp,* to which *eba-175* belongs) and *pfRh* (also known as *pfnbp* or *pfrbl*), and recent data suggest that some members of the two families may have overlapping roles [4]. All individual members of these two gene families seem to be non-essential, as they can be knocked out without impairing parasite growth (reviewed in [2]). AMA1 and thrombospondin repeat domain–containing proteins may also play a role in the formation or migration of the junction [2]. Whatever the precise proteins involved, it is clear that there is redundancy in many of the ligand–receptor interactions between the apical end of the parasite and the erythrocyte. The particular set of receptor–ligand interactions used for invasion determine the so-called alternative invasion pathways, which are described by the sensitivity of invasion to treatment of erythrocytes with various enzymes. It is well established that both field isolates and laboratory-adapted parasite lines vary in their capacity to use the different invasion pathways [5,6].

In the next stage of invasion, the junction migrates towards the posterior end of the parasite driven by a parasite actin-myosin motor, creating an invagination in the erythrocyte membrane. The final step is the sealing of the invagination, which forms a parasitophorous vacuole where the parasite will reside until the next cycle of invasion. Although it is not well established which proteins participate in the formation of the vacuole and remodelling of the erythrocyte, it is possible that the high molecular mass rhoptry complex (RhopH complex) has a role [7], in addition to a possible role at earlier stages of invasion. Two of the components of this trimeric complex, RhopH2 and RhopH3, are encoded by single copy genes, whereas RhopH1/Clag can be encoded by five different genes of the *clag* gene family [8].

We recently described two essentially isogenic parasite lines both derived from the cloned parasite line 3D7 but maintained independently for several years, 3D7-A and 3D7-B. These two parasite lines differ dramatically in their capacity to use invasion pathways that permit entry into mutant and enzyme-treated erythrocytes. Remarkably, 3D7-A can invade erythrocytes sequentially treated with neuraminidase plus trypsin, which are completely resistant to invasion by 3D7-B and by all other parasite lines tested [9]. We hypothesised that the different invasion phenotypes of 3D7-A and 3D7-B parasite lines might be accounted for by differences in the expression of some invasion-related genes. In order to identify invasion-related genes under variant expression and to identify the genes responsible for the different invasion phenotypes of 3D7-A and 3D7-B, we performed a microarray comparison of the two parasite lines. While we did not identify the genes responsible for the different invasion phenotypes of the two parasite lines, our experiments led to the identification of invasion-related genes that exhibit variant expression. We also analysed the transcription of invasion-related genes in subclones of 3D7-A and demonstrated the epigenetic nature of the silencing observed for several genes.

## Results

### Microarray Experiments and Comparison of EBA-140 in 3D7-A and 3D7-B

**Figure 1:**
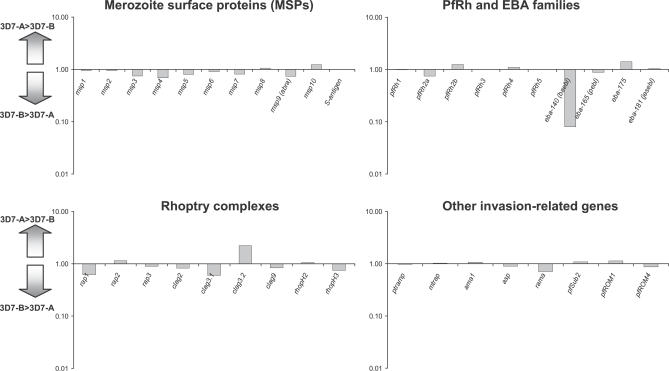
Microarray Comparison of Expression of Invasion-Related Genes between 3D7-A and 3D7-B Schizonts Values correspond to the ratio of expression in 3D7-A versus 3D7-B. Values for each parasite line are the average of two experiments.

Microarray experiments were used to compare tightly synchronised schizonts of the parasite lines 3D7-A and 3D7-B (Dataset S1). After excluding from the analysis all genes from the large *var, rif,* and *stevor* families, which are unlikely to participate in the process of erythrocyte invasion, three genes were found to be expressed at very different levels (more than 5-fold difference) between 3D7-A and 3D7-B: *eba-140* (MAL13P1.60, also known as *baebl*), *pfg27/25* (PF13_0011), and *acylCoA binding protein* gene *(acbp)* on Chromosome 14 (*acbp-14,* PF14_0749). The three genes were expressed at much higher levels in 3D7-B than in 3D7-A, with fold differences of 12.4, 11.2, and 7.7, respectively. Most genes presumed to be involved in erythrocyte invasion were expressed at similar levels between 3D7-A and 3D7-B, with the exception of the aforementioned *eba-140* (see Figure 1 for the best-characterised genes). The 95% identical genes *clag3.1* and *clag3.2* were expressed at different levels in the two parasite lines, but the difference was only about 2-fold. However, the high level of identity between the two genes most likely resulted in cross-hybridization of some of the probes and consequent underestimation of the differences (see below).

**Figure 2:**
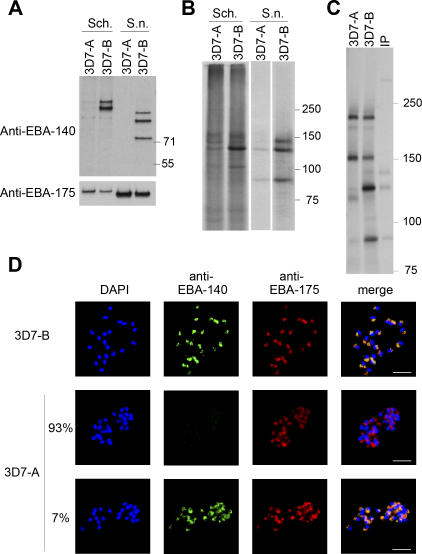
Analysis of EBA-140 in 3D7-A and 3D7-B (A) Western blot analysis of schizonts extracts (Sch.) or culture supernatants (S.n.) probed with rat anti-EBA-140 antibodies. The same membrane was re-probed with rabbit anti-EBA-175 antibodies to control for the amount of stage-specific material. The position of SeeBlue Plus2 pre-stained standards (Invitrogen) is shown (kDa). (B) Immunoprecipitation of NP-40-extracted schizonts and culture supernatants with anti-EBA-140 antibodies. The position of Precision Plus All Blue pre-stained standards (Bio-Rad, http://www.bio-rad.com/) is shown. (C) Erythrocyte binding assay with radiolabelled supernatants from 3D7-A and 3D7-B. The lane IP corresponds to 3D7-B supernatant immunoprecipitated with anti-EBA-140 antibodies. (D) IFA of 3D7-A and 3D7-B schizonts with rabbit anti-EBA-140 and mouse anti-EBA-175 antibodies. Middle and lower panels are representative of 93% and 7% of EBA-175-positive 3D7-A schizonts, respectively. Scale bar = 5 μm.

*eba-140* is the only gene among those expressed at very different levels between the two parasite lines that is known to participate in erythrocyte invasion [10], whereas *pfg27/25* is known to have an important role in gametocyte development [11], and the function of *acbp-14* is not known. We first aimed to determine whether lower *eba-140* transcript abundance in 3D7-A compared to that of 3D7-B resulted in lower abundance of EBA-140 protein. Western blot and immunoprecipitation experiments revealed that EBA-140 was abundant both in schizont extracts and in culture supernatants of 3D7-B, but only present at very low levels in 3D7-A (Figure 2A and 2B). The multiple EBA-140-specific bands in Figure 2A and 2B correspond to proteolytic processing products. Furthermore, erythrocyte binding assays revealed a very different composition of proteins that bind to erythrocytes in supernatants from 3D7-A or 3D7-B (Figure 2C). A band of approximately 175 kDa with an identical mobility to EBA-175 (as determined by western blot on samples run side by side, unpublished data) and a band of approximately 152 kDa were observed for both 3D7-A and 3D7-B, but two strong bands with a mobility identical to two of the EBA-140-immunoprecipitated bands were observed only in 3D7-B.

EBA-140 was present by immunofluorescence assay (IFA) in the apical tip of 100% of EBA-175-positive segmented schizonts and free merozoites in 3D7-B, where the two proteins co-localised. The pattern was consistent with the previously described micronemal location for both proteins [10]. In contrast, only a very low percentage (about 7%) of EBA-175 positive schizonts were positive for EBA-140 in 3D7-A (Figure 2D).

### Comparison of Radiolabelled Supernatants from 3D7-A and 3D7-B

**Figure 3:**
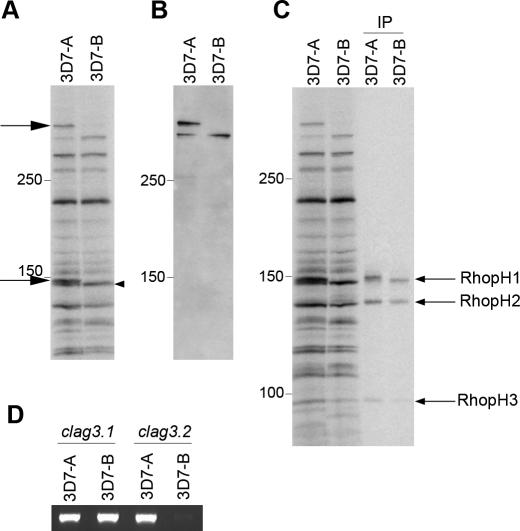
Comparison of Proteins Secreted to the Culture Supernatant between 3D7-A and 3D7-B (A) Radiolabelled culture supernatants run on 20-cm-long 6% SDS-PAGE. The arrows indicate bands present in supernatants from 3D7-A but not from 3D7-B. The arrowhead indicates the band that forms a doublet with the 148-kDa band present only in 3D7-A. (B) Western blot of two lanes identical to those in (A) and run contiguously, probed with rabbit anti-PfRh2b antibodies. (C) Culture supernatants run side by side with identical supernatants immunoprecipitated with the monoclonal antibody 61.3 against RhopH2 (lanes IP). The position of the three members of the RhopH complex is indicated. (D) RT-PCR analysis of *clag3.1* and *clag3.*2 in RNAs from 3D7-A and 3D7-B schizonts.

To detect differences in the expression of invasion-related proteins that might have escaped microarray analysis, we compared radiolabelled culture supernatants from 3D7-A and 3D7-B by SDS-PAGE. Two abundant bands were present in 3D7-A but not in 3D7-B (Figure 3A). A very high molecular mass band corresponds to a form of PfRh2b with an insertion that is present only in 3D7-A [12], as demonstrated by western blot with anti-PfRh2b antibodies on samples run side by side (Figure 3B). The other band has an electrophoretic mobility of approximately 148 kDa and forms a doublet with a band of slightly higher mobility. Because the size of these bands is similar to the size of RhopH1/Clag, we immunoprecipitated supernatants of the two lines with an anti-RhopH2 monoclonal antibody that immunoprecipitates the whole RhopH complex. The immunoprecipitated complex contained an additional band in 3D7-A with an identical mobility to the band present in supernatants of 3D7-A but not of 3D7-B, indicating that the protein present only in 3D7-A supernatants is part of the RhopH complex (Figure 3C). Mass spectrometry analysis revealed the identity of this polypeptide as Clag3.2 (PFC0110w) (25% coverage), whereas the lower band of the doublet (also present in 3D7-B, arrowhead in Figure 3A) was identified as Clag3.1 (PFC0120w) in both parasite lines (33% coverage). Despite the 95% identity between the two proteins, the identification was unambiguous because it was based on three peptides that were specific for one or the other protein (Table 1). As expected from these results, reverse transcriptase (RT)-PCR analysis revealed that *clag3.1* transcripts are present at similar levels in 3D7-A and 3D7-B schizonts, but *clag3.2* transcripts are almost absent in 3D7-B (Figure 3D).

**Table 1:**
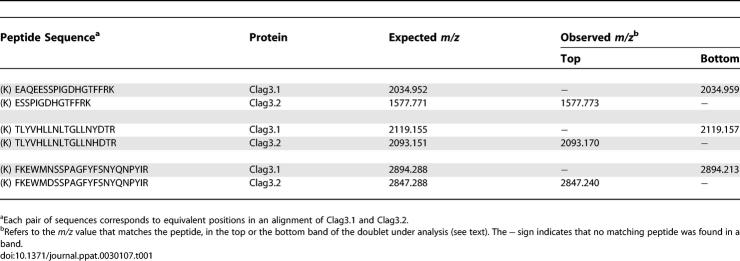
Mass Spectrometry Identification of Clag3.1 and Clag3.2

### Expression of Invasion Ligands in Subclones of 3D7-A

**Figure 4:**
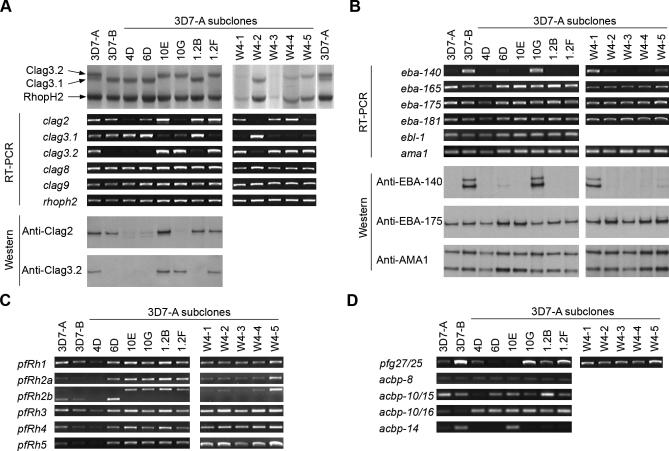
Expression of Invasion-Related Genes in Subclones of 3D7-A (A) Analysis of expression of the *rhoph1*/*clag* family. Top, silver staining of RhopH components in concentrated, Albumax-free culture supernatants from 3D7-A, 3D7-B, and 11 subclones of 3D7-A resolved in 20 cm 6% SDS-PAGE. Middle, RT-PCR analysis from RNA of tightly synchronised schizonts. cDNA samples were the same across the four panels. The single copy *rhoph2* gene was used to control the amount of stage-specific cDNA. The intensity of bands in left and right columns cannot be directly compared (in any of the panels) because they correspond to separate experiments. Bottom, same samples as in the top panel analysed by western blot with anti-Clag2 and anti-Clag3.2 antibodies. (B) Analysis of expression of members of the *eba* family. Top panels, RT-PCR analysis. The single-copy *ama1* gene was used to control the amount of stage-specific cDNA. Lower panels, western blot analysis of schizont extracts with rat anti-EBA-140 antibodies. The same membranes were probed with rabbit anti-EBA-175 and mouse anti-AMA1 antibodies. (C) RT-PCR analysis of expression of genes of the *pfRh* family. The timing of expression of these genes is similar to that of *ama1;* thus, RT-PCR analysis of this gene in (B) controls the amount of stage-specific cDNA. (D) RT-PCR analysis of genes expressed at very different levels between 3D7-A and 3D7-B, and of other members of the *acbp* gene family: *acbp* gene in Chromosomes 8 (PF08_0099), 10 (PF10_0015 and PF10_0016), and 14 (PF14_0749). See Text S1 for the control of stage-specific cDNA.

To determine whether further heterogeneity in the expression of invasion-related genes occurs in the cloned parasite line 3D7, we analysed expression of these genes in 11 subclones of 3D7-A (described in Materials and Methods). Silver-stained SDS-PAGE analysis of culture supernatants from these 11 subclones revealed that all of them expressed either Clag3.1 or Clag3.2, but none of them expressed both (Figure 4A, top panels). Each subclone had gone through at least 11 cycles of replication from a single parasite to harvesting for analysis. The subclones reflect the clonally transmitted expression pattern of the individual parasites from which they originated. This result indicates that 3D7-A is a mixture of parasites expressing one or the other protein. Mutually exclusive expression of the two genes was confirmed by semi-quantitative RT-PCR (Figure 4A, middle panels). All clones that expressed *clag3.1* at high levels had only low-level expression of *clag3.2* and vice versa. Furthermore, another member of the *clag* family, *clag2,* also showed clonal variant expression between the subclones, whereas *clag8* and *clag9* were expressed at very similar levels in all subclones (Figure 4A). Western blot analysis with antibodies specific for Clag2 and Clag3.2 confirmed that low levels of transcripts resulted in low abundance or absence of the corresponding proteins in culture supernatants (Figure 4A, bottom panels). Expression patterns remained stable over continuous culture for at least one additional month (Figure S1A). Interestingly, a stock of the cloned line HB3 at Ehime University (Japan) derived from HB3B, which had been passed through chimpanzees [13], expressed only *clag3.1,* but a stock at the same university derived from HB3A (prior to chimpanzee passage) expressed only *clag3.2,* supporting the idea of mutually exclusive expression and switching between the two genes (Figure S2). HB3 at the National Institute for Medical Research (United Kingdom) and W2mef lines only expressed *clag3.1* at detectable levels (unpublished data).

We also analysed by RT-PCR the expression of members of other multigene families involved in erythrocyte invasion. All members of the *eba* family were expressed at similar levels in the 11 3D7-A subclones except for *eba-140,* which was silenced in most subclones but expressed at levels similar to that of 3D7-B in two of them (Figure 4B). Western blot analysis of schizont extracts revealed that abundance of EBA-140 protein correlated well with transcript abundance (Figure 4B, bottom panels). The pattern of expression of EBA-140 in 3D7-A subclones was fully consistent with the result of IFA experiments (Figure 2D).

Expression of some members of the PfRh family had previously been described as varying between non-isogenic parasite lines [14–16]. We found only small differences in the level of expression of these genes among our subclones, with the exception of *pfRh2b* that was expressed at low levels in the subclones 4D and W4–1 to W4–4 (Figures 4C and S1B). See Text S1 for an explanation of the confounding effect of small differences in the stage of the parasites and the controls developed to overcome this difficulty.

Variant expression among subclones was also observed for two genes, *pfg27/25* and *acbp-14,* which were expressed at higher levels in 3D7-B than in 3D7-A according to the microarray analysis (Figure 4D). Analysis of the subclones revealed that these genes are also silenced in some individual parasites but expressed at levels similar to that of 3D7-B in others. *acbp-14* belongs to a four-gene family [17], but expression of the other genes of this family was similar in all 3D7-A subclones (Figure 4D and Text S1).

### Invasion Phenotype of 3D7-A-Derived Parasite Lines That Differ in the Expression of Invasion Ligands

**Figure 5:**
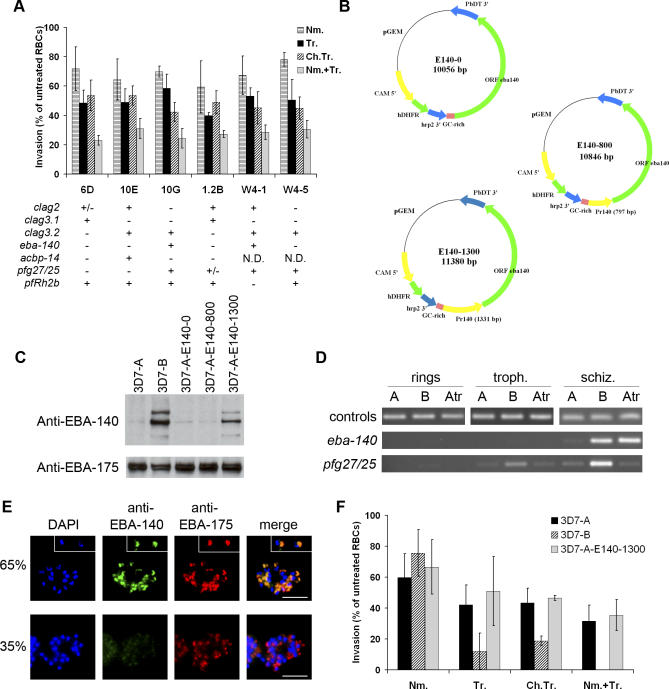
Invasion Phenotypes Associated with the Expression Status of Variantly Expressed Invasion-Related Genes (A) Invasion phenotype of 3D7-A subclones. Values correspond to percent of invasion into untreated erythrocytes and are the average of three to four independent experiments, with 95% confidence interval. Expression of genes that vary in expression between the subclones is shown underneath. The signs + and − correspond to high or low expression relative to other subclones. N.D., not determined; Nm., neuraminidase; Tr., trypsin; Ch.Tr, chymotrypsin; Nm.+Tr, neuraminidase plus trypsin. (B) Scheme of the plasmids transfected in 3D7-A parasites for the episomal expression of EBA-140. (C) Western blot analysis of schizont extracts, as in Figure 2A. Lanes are 3D7-A, 3D7-B, or 3D7-A parasites transfected with the different plasmids, as indicated. (D) RT-PCR analysis at different stages of the life cycle in 3D7-A (lanes A), 3D7-B (lanes B), and E140-1300-transfected 3D7-A (lanes Atr). The amount of stage-specific cDNA was controlled by amplification of the genes PF13_0275 (rings), PF10_0121 (trophozoites), and *ama1* (PF11_0344) (schizonts). (E) IFA of E140-1300-transfected 3D7-A schizonts. Top and lower panels are representative of 65% and 35% of EBA-175-positive schizonts, respectively. The inserts in the top panels are free merozoites. Scale bar = 5 μm. (F) Invasion phenotype of E140-1300-transfected 3D7-A parasites compared to that of 3D7-A and 3D7-B. Values correspond to percent of invasion into untreated erythrocytes and are the average of two independent experiments, with 95% confidence interval.

Despite differences in the expression of several invasion-related proteins, all the 3D7-A subclones tested had very similar growth rates as determined in a one-cycle FACS-based growth assay (Table 2). Likewise, the capacity to invade erythrocytes treated with various enzymes was very similar among all the 3D7-A subclones tested and indistinguishable from that of the parental 3D7-A, but different from that of 3D7-B (see Figure 5A and Figure 5F for comparison). All the 3D7-A subclones invaded erythrocytes sequentially treated with neuraminidase plus trypsin, which are completely resistant to invasion by 3D7-B. Thus, the expression status of *clag2, clag3.1, clag3.2, eba-140, acbp-14, pfg27/25,* or *pfRh2b* did not affect the invasion pathways used by the parasites. This was confirmed by selection-based experiments (Figure S3).

**Table 2:**
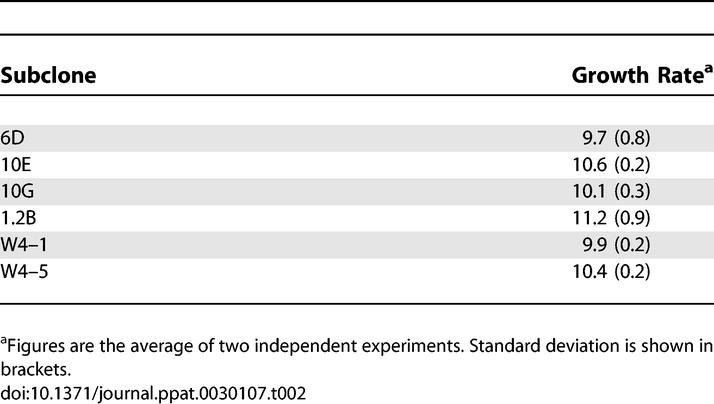
Growth Rate of 3D7-A Subclones

We used an additional approach to confirm that silencing of *eba-140* does not alter the invasion phenotype of the parasites and is not necessary for the invasion of neuraminidase plus trypsin–treated erythrocytes. We expressed *eba-140* in 3D7-A parasites under the control of its own promoter on an episome. We tried three different constructs that contained 0, 797, or 1,331 bp of the region upstream from the *eba-140* start codon to drive the expression of the episomal transgene (Figure 5B). Transfection with these constructs resulted in production of EBA-140 protein only when the longer version of the 5′ region was used (E140-1300), as determined by western blot (Figure 5C). The timing of expression of the episomal *eba-140* was correct, because transcripts of this transgene were undetectable by RT-PCR in ring or trophozoite stages, but were highly abundant in schizonts, as observed for authentic *eba-140* in 3D7-B (Figure 5D). Furthermore, episomally expressed EBA-140 co-localised with EBA-175 by IFA (Figure 5E), indicating that it is correctly located in the apical organelles. Altogether, these results indicate that this large protein can be correctly expressed from an episome. However, only 65% of EBA-175-positive schizonts were EBA-140 positive, probably due to defective segregation of the episome.

E140-1300-transfected 3D7-A parasites had an invasion phenotype indistinguishable from that of 3D7-A, but clearly distinct from that of 3D7-B (Figure 5F). Despite expressing EBA-140, these parasites invaded erythrocytes double-treated with neuraminidase plus trypsin as efficiently as 3D7-A parasites, in contrast to 3D7-B parasites that completely failed to invade them. Invasion assays were performed in the absence of drug. To rule out the possibility that only parasites that had lost the episome (and consequently were not expressing EBA-140) were able to invade double-treated erythrocytes, parasites that had invaded these erythrocytes were placed back under drug pressure and found to have survival rates similar to those invading control erythrocytes.

### Chromosomal Structure of *eba-140, clag3.1,* and *clag3.2* Loci in Parasite Lines That Vary in the Expression of These Genes

**Figure 6:**
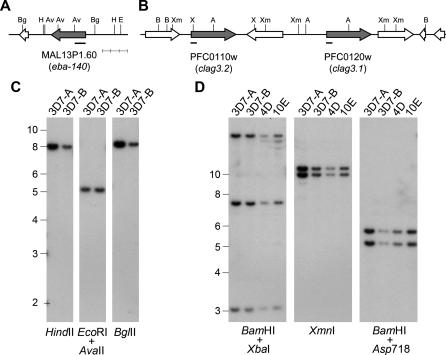
Southern Blot Analysis of *eba-140, clag3.1,* and *clag3.2* Loci (A and B) Genomic organization around the loci under study, showing the restriction sites used for Southern blot analysis and the genes annotated in PlasmoDB (block arrows, genes under analysis in grey, neighbours in white). Diagrams are to scale. The scale bar corresponds to 3 kb. Restriction sites are BglII (Bg), HindII (H), AvaII (Av), EcoRI (E), BamHI (B), XmnI (Xm), Asp718 (A), and XbaI (X). The short lines underneath the genes indicate the position of the probes used. (C and D) Southern blot analysis. The probes used were a 1,304-bp BglII-AvaII restriction fragment from the E140–0 plasmid corresponding to the beginning of the *eba-140* ORF (C) or a 1:1 mixture of PCR-amplified fragments of about 700 bp corresponding to the beginning of the ORF of *clag3.1* and *clag3.2* (D). The position of DNA size markers is indicated (kb).

To determine whether there were genetic differences in the *eba-140, clag3.1,* or *clag3.2* genes associated with their expression status, we analysed these loci in parasite lines where the genes were either expressed or not. Southern blot analysis of the *eba-140* locus, covering the open reading frame (ORF), and also 4.1 kb upstream from the start codon and 3.8 kb downstream from the stop codon, did not reveal any difference between 3D7-A and 3D7-B parasites (Figure 6A and 6C). Furthermore, an *eba-140*-specific PCR product was amplified from genomic DNA from all 3D7-A subclones, including those that do not express the gene. Similarly, Southern blot analysis of the chromosomal region where *clag3.1* and *clag3.2* are located, including the region between the two genes and 2.9 kb upstream from the start codon of *clag3.2* and 6.4 kb downstream from the *clag3.1* stop codon, did not reveal any difference between parasite lines that only express *clag3.1* at high levels (3D7-B and 4D), a parasite line that only expresses *clag3.2* (10E), and 3D7-A, which is a mixture of parasites that express one or the other gene (Figure 6B and 6D). Altogether, these results rule out the possibility that low expression of *eba-140, clag3.1,* or *clag3.2* was associated with major chromosomal rearrangements, deletions, or recombination of the two *clag* genes in Chromosome 3 to form a single gene.

Furthermore, direct sequencing of PCR products spanning the ORF of *eba-140* and 1.3 kb upstream from its start codon did not reveal any difference between 3D7-A and 3D7-B. We also PCR amplified the full *clag3.1* and *clag3.2* ORFs in these two parasite lines with primers in their divergent 5′ and 3′ UTR sequences. Sequencing of these PCR products revealed no difference between the two parasite lines or compared with the published sequences for the genes, which rules out the possibility that a gene conversion event had occurred.

### Transcriptional Analysis of the Left Subtelomeric Region of Chromosome 13

**Figure 7:**
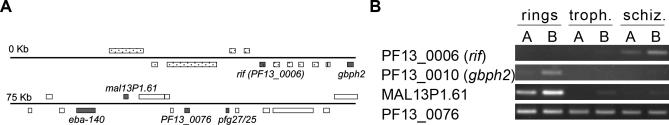
Expression of Genes Located in the Left Subtelomeric Region of Chromosome 13 (A) Schematic of the genes in the left end of Chromosome 13, as annotated in PlasmoDB. Genes above the line are transcribed towards the centromere, whereas genes below the line are transcribed towards the telomere. The genes analysed by RT-PCR are highlighted in grey, with their names. Members of the *var, rif,* and *stevor* families are spotted. (B) RT-PCR analysis of RNA from rings, trophozoites, or schizonts of 3D7-A (lanes A) and 3D7-B (lanes B). The cDNA preparations used were the same as in Figure 5D. Thus, the controls for the amount of stage-specific cDNAs in Figure 5D also apply here.

The two genes expressed at most different levels between 3D7-A and 3D7-B as determined by microarray analysis, *eba-140* and *pfg27/25,* are located in the left subtelomeric region of Chromosome 13 (Figure 7A), at a distance of 89.4 and 121.8 kb from the telomere, respectively. This is suggestive of coordinated regional silencing of this subtelomeric zone in 3D7-A. None of the genes located between *eba-140* and *pfg27/25* or between the telomere and *eba-140* is expressed at high levels in schizonts [18,19]; thus, our microarray experiments were unable to determine whether they are also differentially expressed between 3D7-A and 3D7-B. We prepared RNA from tightly synchronised ring- (11–16 h), trophozoite- (23–28 h), or schizont-stage parasites (39 h and 30 min to 44h and 30min for 3D7-A, 41–46 h for 3D7-B) and performed RT-PCR analysis of *eba-140, pfg27/25,* and four additional genes located in this chromosomal region (Figures 5D and 7). Two of the genes with a peak of expression in rings, *gbph2* and MAL13P1.61, were expressed at slightly higher levels in 3D7-B than in 3D7-A, and the same was true for the member of the *rif* family, PF13_0006, which was unexpectedly expressed in schizonts. On the other hand, PF13_0076 was expressed at all stages and at similar levels in both parasite lines (Figure 7B). Thus, higher expression in 3D7-B than in 3D7-A occurred for several genes in this chromosomal region, but different genes were affected to different extents. The most marked differences were observed for genes expressed in late schizonts *(eba-140* and *pfg27/25)*.

### In Situ Activation of *eba-140* by Insertion of a Drug Resistance Marker Gene in Its Vicinity

**Figure 8:**
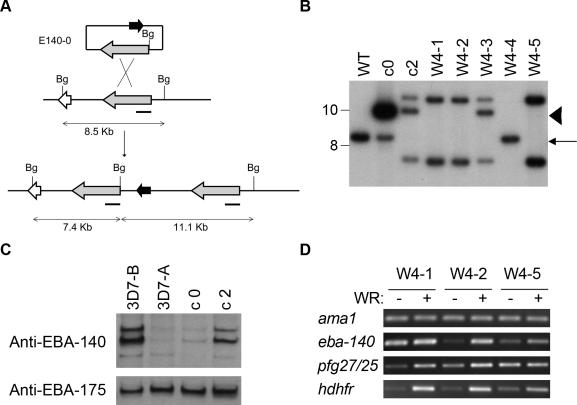
Activation of *eba-140* by Insertion of a Drug Resistance Marker in Its Vicinity (A) Integration of the construct E140–0 (see Figure 5B) into the genomic *eba-140* locus. The *eba-140* and *hdhfr* genes are indicated by grey and black block arrows, respectively. The positions of the BglII sites (Bg) and the probe (line underneath the gene) used for the Southern blot analysis are indicated. (B) Southern blot analysis of E140–0 transfected 3D7-A parasites. The lanes correspond to BglII-digested genomic DNA from wild-type 3D7-A (lane WT), 3D7-A transfected with E140–0 before (lane c0) or after two cycles on/off drug (lane c2), and five subclones of the latter (lanes W4–1 to W4–5). The arrow indicates the position of the band for the wild-type gene and the arrowhead the position of the episome (identical to the position of a band obtained by integration of multiple copies of the construct). (C) Western blot analysis (as in Figure 2A) of schizonts extracts of 3D7-A, 3D7-B, and E140–0-transfected 3D7-A parasites before (lane c0) and after two cycles on/off drug (lane c2). (D) RT-PCR analysis of schizonts from subclones that had been maintained for 1 mo either in the absence (lanes −) or the presence (lanes +) of WR99210 drug (increasing concentrations from 10 to 40 nM). The single-copy gene *ama1* was used to control the amount of stage-specific cDNA.

Integration of the construct E140–0 (Figure 5B) in the *eba-140* locus by a single-recombination event was achieved by cycling E140–0-transfected 3D7-A parasites for two cycles on/off drug and confirmed by Southern blot (Figure 8A and 8B). The integration of the construct resulted in the duplication of the *eba-140* gene, but only one copy was preceded by sequences with promoter activity (Figures 8A and 5B). Western blot analysis revealed that integration of this plasmid resulted in expression of the *eba-140* gene, though at a lower level than in 3D7-B (Figure 8C).

E140–0-transfected and -drug-cycled 3D7-A parasites were subcloned by limiting dilution. Five of the resulting subclones were analysed by Southern blot, which revealed that three of them had integrated one copy of the gene (W4–1, W4–2, and W4–5), whereas one had integrated two or more copies (W4–3) and one was wild type (W4–4) (Figure 8B). Subcloning was performed in the absence of drug pressure. RT-PCR and western blot analysis of these subclones revealed that only one of them (W4–1) expressed EBA-140 at high levels, whereas all the other subclones expressed it at low levels similar to that of the parental 3D7-A (Figure 4B). The three subclones that had one copy of the construct integrated were maintained in parallel either in the absence or presence of drug selection for 1 mo, and RNA from tightly synchronised schizonts of the resulting populations was analysed by RT-PCR. The presence of the drug resulted in a large (W4–1 and W4–2) or moderate (W4–5) increase in the abundance of transcripts of the drug resistance gene *hdhfr*. The increase was paralleled by a dramatic increase in the abundance of *eba-140* transcripts in the clone W4–2, whereas expression of this gene was not affected in the clone W4–1, which already expressed *eba-140* at high levels before drug selection, and only moderately increased in the clone W4–5 (Figure 8D). This result indicates that insertion of a gene *(hdhfr)* that is forced to be active (by drug selection) in the vicinity of *eba-140* can cause the activation of this gene. Thus, *eba-140* can be activated in situ, ruling out the possibility that undetected genetic changes were responsible for the silencing and indicating that silencing was epigenetic. Expression of the gene *pfg27/25,* which is more distal to the telomere than *eba140* and was already active in the absence of drug in the three clones (Figure 4D), was affected to a lower extent (Figure 8D).

## Discussion

The process of erythrocyte invasion by merozoites of P. falciparum involves several essential, highly conserved interactions as well as dispensable, redundant interactions. Here we show that several of the genes responsible for the latter can be epigenetically silenced. Some members of the *pfRh* family had been shown to vary in expression between non-isogenic cloned parasite lines [14,15] and among field isolates [16]. Furthermore, switching from sialic acid–dependent into sialic acid–independent invasion in the two related parasite lines W2mef and Dd2 involved increased expression of PfRh4 [4,20]. Here, we extend the observation of variant expression to several other invasion-related gene families in isogenic parasite lines and demonstrate that silencing is transmitted epigenetically.

The comparison of the two isogenic parasite lines 3D7-A and 3D7-B revealed differences in the expression of *eba-140, clag3.2, pfg27/25,* and *acbp-14*. To determine whether further heterogeneity exists within the cloned line 3D7, we analysed the expression of invasion-related genes in 11 subclones of 3D7-A, which reflect the pattern of expression in the 11 3D7-A individual parasites from which they originated. Clonal variant expression was detected for three additional genes, *clag2, clag3.1,* and *pfRh2b*. In all cases tested, mRNA abundance reflected protein abundance. Thus, 3D7-A is a mosaic of parasites expressing different combinations of invasion proteins. It will be important to determine to what extent this mosaicism occurs in natural parasite populations.

A main feature of all the invasion-related genes showing variant expression is that they belong to small multigene families. The *var* genes, which are the paradigm of variant expression in *Plasmodium,* are also part of a multigene family, though of a much larger size. *var* genes, which participate in both immune evasion and cytoadhesion of infected erythrocytes, exhibit mutually exclusive expression, such that only one gene of the family is expressed at a time [21]. In the case of invasion-related multigene families, we observed mutually exclusive expression for two members of the *clag* family, *clag3.1* and *clag3.2*.

We did not detect any DNA alteration associated with the active or silent state of invasion-related genes. Although we cannot exclude the possibility of minor alterations that escaped our analysis, or that regulatory regions where alterations occurred were located in distant regions of the chromosome, the most plausible explanation is that, at least in the cases of *clag3.1, clag3.2,* and *eba-140,* silencing was transmitted epigenetically. This is again reminiscent of the situation for *var* genes, which switch from a silent to an active state without detectable DNA alterations [21] and which are epigenetically silenced in a process that involves modifications of the chromatin structure [22,23]. Furthermore, approximately two-thirds of *var* genes and the majority of members of small invasion-related multigene families for which we detected variant expression are located in subtelomeric positions (Figure S4), though *var* genes are always more proximal to the telomere. It will be important to determine whether the subtelomeric location of these invasion-related genes is critical for their variant expression or, instead, is related to their evolution.

In addition to the absence of genetic alterations between an active and a silenced state, reversibility and region-specific rather than sequence-specific effects are hallmarks of epigenetic silencing and heterochromatin. Both were demonstrated for the silencing of *eba-140*. Insertion of the drug resistance gene *hdhfr* in the vicinity of the *eba-140* locus and subsequent drug pressure resulted in the in situ activation of this gene in some subclones. This suggests that activation of *hdhfr* disrupted a compact, “closed” conformation of the chromatin around this locus and forced the transition to a more relaxed, transcriptionally active conformation that spread into the neighbour *eba-140*. Furthermore, silencing of *eba-140* in 3D7-A was somehow coordinated with silencing of another gene located in the same subtelomeric region, *pfg27/25*. This locus was silenced in some subclones of 3D7-A but expressed in others, but in all cases where the more telomere proximal *eba-140* gene was active, *pfg27/25* was also active. This suggests a model in which silenced chromatin would spread from a telomeric position. The extent of the silenced area would vary stochastically between individual parasites, but once established it would be clonally inherited, which would explain the variegated expression of the two genes in the different subclones. In some subclones, the silenced area would spread as far into the chromosome as the *pfg27/25* locus, while in others it would only reach *eba-140* but not *pfg27/25,* and in others it would not even reach *eba-140*. However, the observation that other genes located in the same region were only silenced to a lower extent or not silenced at all does not support this model, and suggests that either the silenced chromatin structure is only formed late in the life cycle of the parasite, or a mechanism other than heterochromatin spreading is responsible for the coordinated silencing of these genes. Further experiments will be needed to distinguish between these two possibilities. It will also be important to determine whether transcripts from PF13_0076 in schizonts, which occurred at similar levels in 3D7-A and 3D7-B, represent active transcription at this stage or carry over of mRNA transcribed in previous stages.

In contrast to the apparently region-specific, sequence-independent variegated silencing of the *eba-140* locus, silencing of *clag3.1* or *clag3.2* was promoter specific, because the two genes lie adjacent to each other in the genome and expression was mutually exclusive. This situation is more similar to that observed for *var* genes, where one gene can be activated while its neighbours remain silenced [24]. The chromosomal organization of *clag3.1* and *clag3.2* resembles that of two other invasion-related genes with a high level of identity, *pfRh2a* and *pfRh2b,* which lie adjacent to each other near the centromeric region of Chromosome 13. However, in that case, expression is coordinated rather than mutually exclusive, with all parasite lines analysed so far expressing either none or both of the genes (with the exception of parasite lines in which one of the genes is missing) [14–16,25]. The contiguous genes *PfRh4* and *eba-165* also seem to be co-regulated [4,20].

This complex picture reveals the existence of multiple different ways of regulation of the expression of genes encoding P. falciparum erythrocyte invasion proteins. Although the pattern of silenced and expressed genes may be very different in parasites living in the context of a host with acquired immune responses, our results on culture-adapted parasite lines demonstrate the existence in P. falciparum of the molecular machinery for the silencing of these genes in addition to the life cycle–dependent silencing common to most *Plasmodium* genes [18]. Epigenetically transmitted transcriptional silencing of invasion-related genes together with a certain level of mosaicism in a parasite population provides an enormous flexibility and capacity to adapt rapidly to changing host environments by simple means of natural selection and stochastic, low-frequency switching on and off of the expression of these genes.

The biological role of variant expression of invasion-related genes is unknown, and we can only speculate about its possible functions. While targeted disruption of the invasion-related genes *eba-175* and *pfRh2b* resulted in changes in the invasion pathways used by the parasites where they were disrupted [15,26], the active or silent state of the variantly expressed genes described in this study was not associated with detectable differences in growth rates or in the invasion pathways used. Regardless of the combination of silenced and expressed invasion-related genes, all 3D7-A subclones were able to invade using the neuraminidase- and trypsin-resistant receptor A [9], indicating that none of the genes found to vary in expression is responsible for this interaction. The identical invasion phenotype of all 3D7-A subclones suggests that the main force driving the variant expression of invasion-related genes is not the acquisition of the ability to invade different types of erythrocytes, but instead is immune evasion. This is a reasonable hypothesis, considering that in human populations diversity of the erythrocyte receptors that the parasite uses for invasion is somehow limited, whereas merozoites are exposed to protective immune responses that are extremely diverse between different hosts. However, when silencing affects other invasion-related genes like *eba-175* or occurs in other genetic backgrounds, it will clearly affect the types of erythrocytes susceptible to invasion. Epigenetic silencing of invasion-related genes is likely to be the mechanism behind switching between invasion pathways.

The number of genes in the invasion-related multigene families is small for a role in immune evasion, especially when compared with the size of well-characterised variantly expressed gene families such as the *var* genes in P. falciparum or the *vsg* genes in Trypanosoma brucei. However, all members of the *clag, eba,* and *pfRh* families are polymorphic to some extent, and in some cases polymorphism is the consequence of positive selection [27,28]. Polymorphism, which is well known to help the parasite escape immune responses, and variant expression based on a limited number of alternatives, may play additive or even synergistic roles in immune evasion. Variant expression has the potential to enhance the capacity of polymorphism to avoid immune responses by permitting selection of parasites that keep in a silenced state the multigene family members with polymorphic allelic forms that are better recognised by protective immune responses in a particular host.

In summary, we describe an additional layer of complexity of the process of erythrocyte invasion, showing that the parasite has multiple modes of controlling the expression of genes involved in this process. This may provide an advantage to the parasite in its constant race to escape the immune system of its human host.

## Materials and Methods

### Parasites, transfection, and subcloning.

Parasite cultures were maintained under standard conditions in medium containing Albumax II. The parasite lines 3D7-A and 3D7-B are the same cloned parasite line 3D7 maintained in different laboratories for several years. Genotyping was used to confirm their 3D7 identity [9]. The subclones 4D, 6D, 10E, 10G, 1.2B, and 1.2F were originated by subcloning 3D7-A by limiting dilution [12]. The subclones W4–1 to W4–5 were obtained by subcloning by limiting dilution 3D7-A parasites that had been transfected with the plasmid E140–0 and went through two cycles on/off drug to promote integration of the plasmid (see Figure 8 and Results). Genotyping of the highly polymorphic gene *msp2* by HinfI restriction fragment length polymorphism (RFLP) [29] was used to confirm the 3D7 identity of all subclones and rule out contamination from other parasite lines (unpublished data). Details of the methods used for transfection and subcloning are provided in Text S1.

### One-cycle FACS-based growth assay, erythrocyte digestions, and invasion assays.

To determine the growth rate of different subclones of the parasite line 3D7-A, synchronised cultures of the different subclones were diluted with fresh erythrocytes to an approximate parasitaemia of 0.7% immediately after sorbitol lysis. After culturing for 15 to 20 h, when most of the parasites were at the late trophozoite or early schizont stage, parasitaemia was determined by FACS as described [30] (time 0) using a FACScalibur cytometer (Becton Dickinson, http://www.bd.com/). Parasitaemia was determined again by FACS 48 h later, and the growth rate determined as the ratio between the parasitaemia at the 48- and 0-h time points. Erythrocyte digestions and invasion assays were performed as described [9], with small modifications explained in Text S1.

### Preparation of RNAs, reverse transcription, and semi-quantitative PCR.

To obtain RNA for microarray or RT-PCR analysis, parasites were synchronised to a 5-h window by purifying schizonts from a culture with abundant late forms on 70% Percoll, and removing late forms by sorbitol lysis 5 h later. Parasites were then left undisturbed for 39 h and 30 min (3D7-A and subclones) or 41 h (3D7-B), and harvested in 20-erythrocyte pellet volumes of Trizol (Invitrogen, http://www.invitrogen.com/). These times were determined in preliminary experiments for each parasite line as the times at which 20% of the schizonts had burst (estimated from the ratio of rings to schizonts). RNA in Trizol was frozen at −70 °C and later purified according to the manufacturer's instructions. RNA was then treated with RNAse-free DNAse I (Qiagen, http://www.qiagen.com/) and cleaned with the RNeasy MinElute cleanup kit (Qiagen). cDNA was obtained by reverse transcribing 0.5 μg of total RNA using the AMV reverse transcription kit (Promega, http://www.promega.com/) with oligo dT primers. To rule out the possibility of gDNA contamination, parallel reactions were performed for all samples in the absence of reverse transcriptase and tested by PCR with at least two primer pairs. To achieve semi-quantitative conditions, PCR was performed for only 25 cycles, and the amount of starting cDNA was adjusted for each primer pair to obtain bands that were clearly visible but not saturating. Single-copy genes with similar timing of expression [18,19] to the genes under analysis were used to control the amount of cDNA specific of each stage. All of the primers used in this study are described in Dataset S2.

### Microarray analysis.

The Affymetrix PFSANGER array (http://www.affymetrix.com/) was used for these experiments. Details of the array, experimental procedure, RMA normalization, and data analysis are available in Text S1.

### Plasmids and antibodies.

The procedure used for the construction of the plasmids E140–0, E140–800, and E-140-1300 (Figure 5B) is explained in Text S1. The antibodies used in this study and their sources are also described in Text S1.

### Preparation of schizont extracts, culture supernatants, and metabolic labelling of parasites.

Schizont extracts for western blot were prepared by resuspending pellets of Percoll-purified schizonts into 20-pellet volumes of PBS, adding 40-pellet volumes of 2x SDS protein loading buffer and heating for 5 min at 95 °C before storing at −70 °C until use. NP-40 extracts of schizonts for immunoprecipitation were prepared approximately as described [14]. To obtain culture supernatants, tightly synchronised parasite cultures with abundant segmented schizonts were enriched for schizont-infected erythrocytes by gelatin flotation. Schizont-enriched fractions (typically 80% parasitaemia) were placed back in culture at a haematocrit of approximately 0.3% and supernatants harvested by centrifugation 13 to 20 h later. For the preparation of supernatants in Albumax-free medium, it was critical that the original culture only contained very mature forms, because otherwise it resulted in death of the parasites before rupture and under-representation of some of the proteins usually released into the culture supernatant (unpublished data). Metabolic labelling of parasites was achieved approximately as described [14].

### Fractionation of culture supernatants and mass spectrometry.

Concentrated culture supernatants (from 5 ml of original supernatant) prepared in the absence of Albumax were loaded into a Q-Sepharose column in 0.5x PBS buffer. After washing with 0.5x PBS + 0.1 M NaCl, elution was performed with 0.5x PBS + 0.25 M NaCl. This fractionation resulted in a significant enrichment in the proteins of interest and elimination of haemoglobin. For mass spectrometry, fractions containing the proteins of interest were concentrated and resolved in 20 cm 5.5% polyacrylamide gels. The bands of interest were excised, reduced, alkylated, and trypsin-digested, then the released peptides were processed for mass spectrometry and analysed in a Reflex III MALDI-ToF mass spectrometer (Bruker Daltonics, http://www.bdal.de/). Data were analysed using Mascot software (Matrix Science, http://www.matrixscience.com/). Analysis of an excision in the 3D7-B lane corresponding to the position of Clag3.2 yielded no signal.

### SDS-PAGE, western blot, immunoprecipitation, erythrocyte binding assays, and immunofluorescence assays.

Details of the procedures used for these experiments are available in Text S1.

## Supporting Information

Dataset S1 Microarray Comparison of 3D7-A and 3D7-B Expression values for the 5,685 genes of P. falciparum analysed on the PFSANGER microarray, normalised, and logged (base 2) values (columns 2–5). The values provided for each gene (in rows) are the Log2 Ratio (column 6) of the contrast 3D7-B versus 3D7-A, followed by the moderated *t*-stats (column 7 =t), *p*-values (column 8 = P.Value), adjusted *p*-values for multiple correction (column 9 = adj.P.Value), and B statistics or log Odds (column 10 = B) as given by the function “topTable” in the Limma package of Bioconductor (http://www.bioconductor.org/packages/1.9/bioc/html/limma.html).
(1.1 MB XLS)

Dataset S2 Details of the Primers Used in This Study PlasmoDB accession numbers (systematic gene names/IDs) for the genes mentioned in this article are also described in this dataset.
(28 KB XLS)

Figure S1 RT-PCR Analysis of 3D7-A Subclones (A) Stability of the patterns of expression of *clag* genes. RNA from tightly synchronised schizonts of the subclones W4–1, W4–2, and W4–5 was prepared after maintaining the parasites in culture for an additional 32 d after they were harvested to prepare the RNA used in Figure 4. The pattern of expression remained identical (compare with Figure 4A). These parasite lines had been cultured for almost 2 mo since cloning, indicating that silencing or expression of these genes is stably clonally transmitted for at least 25–30 cycles of division.
(B) RT-PCR analysis of *pfRh* genes in two subclones of each of the two groups that were analysed in separate experiments in Figure 4. Expression can be compared here among these four subclones. W4–1 and W4–2 (and by extension W4–3 and W4–4) express lower amounts of *pfRh2b* than 1.2B and 1.2F (and by extension 6D, 10E, and 10G), whereas expression of other members of the *pfRh* multigene family was similar among all the subclones.
(92 KB TIF)

Figure S2 Quantitative PCR Analysis of Expression of *clag3.1* and *clag3.2* in HB3 Parasites P. falciparum HB3B line was derived from the cloned line HB3A by infecting chimpanzees through mosquito bite [13]. Both clones were obtained from David Walliker. It is important to note that parasite lines 3D7-A and 3D7-B [9] are unrelated to 3D7A and 3D7B described by Walliker and collaborators [13] and were given the same names by mistake. HB3B#1 and HB3B#2 correspond to stocks of HB3B produced on different dates. The subclones C6, F4, F5, and G11 were obtained by limiting dilution of HB3B#2. Real-time RT-PCR analysis was performed with QuantiTect SYBR Green (Qiagen) approximately as described [8] using RNA from relatively synchronised cultures harvested at the schizont stage. Results are expressed relative to expression of *rhoph2,* which is expressed with a similar timing.
pGEM-T easy plasmids containing each DNA fragment were used as standards. The amount of each plasmid standard was compensated by evaluating the relative amount using primers located on the plasmid backbone, SP6 primer (ATTTAGGTGACACTATAGAA) and pGEM.rtF (GCAGGTCGACCATATG). Primers for *clag3.1* and *clag3.2* were described previously [8]. Primers for *rhoph2* were GTAACAACACTTACTAAGGCAGACT and GTACAAAGCTACAATATTGTTAGATCT.
To rule out the possibility that gene conversion had occurred in some of the HB3-derived lines, the full ORF of *clag3.1* and *clag3.2* were PCR amplified from gDNA of HB3A and HB3B with primers located in the divergent 5′ and 3′ UTR regions and the regions used for quantitative PCR sequenced from these PCR products. The results ruled out the possibility that gene conversion was confounding the expression results (unpublished data).
(39 KB TIF)

Figure S3 RT-PCR Analysis of RNA from Parasites Selected for Growth in Different Erythrocyte Types To test the possibility that expression or silencing of certain invasion-related genes might provide only a small selective advantage for the invasion of neuraminidase plus trypsin–treated erythrocytes that escaped detection by standard invasion assays, we selected 3D7-A parasites for growth in these erythrocytes. Furthermore, to test the possibility that the expression status of these genes affected the capacity to invade erythrocytes of different ages, we selected 3D7-A parasites for growth in ageing erythrocytes.
3D7-A parasites were grown in parallel in untreated (lanes Unt.) or neuraminidase plus trypsin–treated (lanes Nm.+Tr.) erythrocytes for four cycles and in fresh (stored for less than a week, always at 4 °C) or ageing (kept for at least one week at room temperature) erythrocytes during eight cycles. Parasites were then grown in fresh untreated erythrocytes to obtain enough parasite material for RNA isolation. RNA from tightly synchronised schizonts was obtained and the expression of invasion-related genes tested by semi-quantitative PCR. The single-copy genes *rhoph2* and *ama1* were used to control the amount of stage-specific cDNA as in Figure 4.
This analysis did not reveal any difference in the balance between *clag3.1* and *clag3.2* or in the expression of any of the other genes tested between the selected and control cultures, with the exceptions of *pfRh2b* and *pfRh4,* which were expressed at lower levels in cultures grown in ageing erythrocytes. This result suggests that these ligands may not be used to invade senescent erythrocytes.
Direct invasion assays did not reveal any major difference among 3D7-A subclones in their capacity to invade ageing erythrocytes, which were invaded at around 30% the rate for fresh erythrocytes (unpublished data).
(208 KB TIF)

Figure S4 Chromosomal Position of Invasion-Related Multigene Families Information was obtained from PlasmoDB (http://www.plasmodb.org/). The position of *clag8* (MAL7P1.229) is indicated with a question mark because it is located in the left subtelomeric region of Chromosome 7 according to PlasmoDB but was experimentally assigned to Chromosome 8 [8]. The majority of genes of invasion-related multigene families that exhibited variant expression (*eba, pfRh,* and *clag* families) are located in subtelomeric positions, with the exception of *pfRh2a* and *pfRh2b,* which are located relatively near the centromere. The *acbp* multigene family, of which one gene was also found to vary in expression but for which a role in the process of invasion has not been established, is also located in subtelomeric positions, with the exception of the *acbp* gene in Chromosome 8.
(21 KB TIF)

Text S1 Supporting Text Online supporting materials and methods, discussion, and references.
(36 KB PDF)

### Accession Numbers

Microarray data have been deposited with ArrayExpress under accession number E-SGRP-9. The PlasmoDB (http://www.plasmodb.org/plasmo/home.jsp) accession numbers (systematic gene names/IDs) for the genes mentioned in this article are described in Dataset S2.

## Abbreviations

IFA: immunofluorescence assay
ORF: open reading frame
RT-PCR: reverse transcriptase PCR
