# Supplementary material for: Epigenetic Silencing of Plasmodium falciparum Genes Linked to Erythrocyte Invasion
Source: PLoS Pathog. 2007 Aug 3;3(8):e107. doi: 10.1371/journal.ppat.0030107 (PMC1937010; doi:10.1371/journal.ppat.0030107)
Supplement: Supplementary file 1 [file ppat.0030107.sd003.pdf]

SUPPORTING TEXT.

Supporting Materials and Methods.

Transfection and subcloning.

Transfection was performed by electroporation of cultures containing abundant ring-stage parasites essentially as described [1], using at least 100 µg of plasmid. The drug WR99210 was added at a 20 nM concentration after 15 to 20 hours. Cultures were maintained at 3% hematocrit, adding fresh medium frequently and diluting with fresh erythrocytes (1:2) every 6 days until parasites were observed, typically around day 14. To induce integration of the plasmid, parasites were subjected to two cycles on/off drug. After analyzing the resulting population by Southern blot (performed according to standard procedures), the culture was subcloned by limiting dilution in 96-well plates by diluting parasites to an expected average of 0.3 parasites per well, keeping the haematocrit at 3% and diluting with fresh erythrocytes after 7 days. Using this procedure, parasites were typically observed in less than 30% of the wells at day 12.

Erythrocyte digestions and invasion assays.

Erythrocyte digestions were performed as described [2], but Calbiochem neuraminidase cat. 480717 was used because the product used in the previous studies (cat. 480700) had been discontinued. This may explain the higher efficiency of invasion of neuraminidase-treated erythrocytes by 3D7-A and especially by 3D7-B compared with our previous studies [2]. Alternatively, the discrepancy may relate to small differences in the culture methods of different laboratories. Invasion assays were always performed in

parallel for all the subclones of 3D7-A under analysis or for 3D7-A, 3D7-B and transfected 3D7-A parasites.

Microarray analysis.

Affymetrix PFSANGER arrays are high-density 8-µm custom 25-mer oligonucleotide arrays, whose tiling-like design was based on the *P. falciparum* genomic sequence released in January 2005 (www.genedb.org). The arrays are PM-only (perfect match) and comprise 2.44 million (M) *Plasmodium* probes, of which 2.32 M are unique and specific to *P. falciparum.* Due to specificity/isothermal constraints during design, the probes are distributed non-randomly throughout the genome. Both strands are represented, with 1.7 M probes in non-coding regions of the genome, and 0.5 M within introns.

Total RNA was reverse transcribed and biotion-labelled as cRNA, using the GeneChip IVT Labelling kit as recommended by Affymetrix. Hybridizations were carried out at 45°C for 16 hr with constant rotation at 60rpm. Following hybridization, the solutions were removed and the arrays washed and stained on a fluidics station (Affymetrix FS450). Gene arrays were then scanned at an emission wavelength of 570nm at 1.56µm pixel-resolution using a confocal scanner (Affymetrix GeneChip Scanner 3000 7G). After scanning, the hybridization intensity for each 25-mer feature was computed using Affymetrix GCOS v1.3 software, and the CEL files transferred into the R/Bioconductor environment ([3]; www.bioconductor.org; www.r-project.org) for downstream analyses, using a coding-sequence predicted loci chip definition file (CDF) generated in-house for these analyses (affy, makecdfenv, altcdfenv packages; [4]). Arrays

were background adjusted and quantile normalised using the robust multiarray averaging algorithm (RMA) [5]. Fold-differences might have been underestimated because RMA normalization assigns relatively high values to genes expressed at background or near-background levels. Differential expression between conditions (contrasts) was estimated by the application of linear models, with Bayesian correction, via the "limma" Bioconductor package. Log2 ratios ("coefficients" or M values) were generated and corrected for false discovery rate using the Bonferroni-Hochberg method [6,7] with associated measures of statistical significance, for each locus in 3D7-B *vs*. 3D7-A contrast.

Plasmids.

The constructs E140-0, E140-800 and E140-1300 (Fig. 5B) were based on pHH1 [1]. Two overlapping fragments spanning the full coding sequence (including introns) of *eba-140* were PCR-amplified from genomic DNA with primers ORF140f1*Bgl* plus ORF140r1 and ORF140f1.5 plus ORF140r2*Xho* using *Pfu* Ultra hotstart polymerase (Stratagene). A mixture of the two fragments was used as a template to PCR-amplify the full gene with primers ORF140f1*Bgl* and ORF140r2*Xho*. This fragment was cloned into *Bgl*II-*Xho*I sites of pHH1, which removed the *P. falciparum* heat shock protein 86 promoter region (*hsp86* 5') previously cloned in this vector. Because it was impossible to clone the *eba-140* promoter region into the resulting plasmid, a 230 bp fragment of unrelated sequence (amplified from the *lacI* gene in the pET-30 vector [Novagen] with primers PET30f*BamEcoCla* and PET30r*AspSpeBgl*) with a G+C content of 58% was cloned between the *eba-140* coding sequence and the drug resistance cassette using

*Bam*HI/*Bgl*II and *Bgl*II sites and creating a new *Eco*RI and other restriction sites, and then the drug resistance cassette was flipped around using two *Eco*RI sites. In addition to making the plasmid more stable and permitting the cloning of a long stretch of promoter region, this prevented expression of *eba-140* with an incorrect timing under the bidirectional activity of the *P. falciparum* Calmodulin (CAM) promoter [8]. The region immediately upstream the *eba-140* start codon (797 or 1331 bp) was amplified with forward primers Pr140f3*Asp* and Pr140f2*Asp*, respectively, and reverse primer Pr140r*Bam*, using Accuprime polymerase (Invitrogen). These fragments were cloned into *Asp*718 and *Bam*HI/*Bgl*II restriction sites. All primers used are described in the Supporting Dataset S2.

Antibodies.

A rat polyclonal antibody against region II of EBA-140 [9] and a rabbit polyclonal antibody against the second half of the first DBL domain of region II of EBA-140 [10] were kind gifts of D.C.G. Mayer and C.-A. Lobo, respectively. The rat and the rabbit antibody were preferentially used for Western blot and for IFA, respectively, because they showed less cross-reactivity. Despite being less cross-reactive, the rat antibody recognized some very high molecular mass bands by Western blot, clearly distinguishable from the EBA-140-specific signal. This antibody was always used on samples separated under non-reducing conditions. Mouse [11] and Rabbit (MRA-2, MR4, ATCC, Manassas, Virginia, USA) polyclonal antibodies against region VI of EBA-175 were a kind gift from M.J. Blackman or obtained from the malaria research and reference reagent resource (MR4), respectively. The polyclonal mouse antibody against

AMA-1 was a kind gift from C.R. Collins. The monoclonal antibody 61.3 against RhopH2 has been described before [12]. A polyclonal rabbit antibody against PfRh2b was a gift from J.W. Barnwell [13]. The polyclonal rat antibody against Clag2 has been described before [14]. The polyclonal mouse antibody against Clag3.2 was raised against a GST-fusion protein containing the part of Clag3.2 that differs the most from Clag3.1 (residues 1115–1148, CAFDPKRCTPDCKNSTSYKSPQSFFYGWPPSSET). Our experiments with 3D7-A subclones demonstrated that in spite of the 95% identity between Clag3.1 and Clag3.2, the antibody is highly specific for Clag3.2 by Western blot.

SDS-PAGE, Western blot, immunoprecipitation, erythrocyte binding assays and immunofluorescence assays.

SDS-PAGE was performed on NuPAGE 3-8% Tris-acetate pre-cast gels (Invitrogen) for regular Western blots or on 20 cm long Tris-glycine gels when better resolution was necessary. Western blot was performed on nitrocellulose membranes following standard procedures. To control for equal loading of stage-specific parasite material between different samples, membranes were stripped with Restore stripping buffer (Pierce) according to the manufacturer's instructions and re-probed with antibodies against proteins that are not expected to vary in expression. Immunoprecipitation of radiolabelled NP-40-extracted schizonts or culture supernatants was performed approximately as described [15]. Erythrocyte binding assays were performed by incubating 0.5 ml of radiolabelled culture supernatant with 0.1 ml of washed erythrocytes for 45 min at 37ºC. After spinning twice through silicon oil (Dow Corning 550, BDH)

and washing with RPMI 1640-HEPES, bound proteins were eluted by incubating with 20 µl of 1.5 M NaCl for 15 min at RT and separated from erythrocytes by centrifugation. Immunofluorescence assays (IFA) were performed on air-dried smears fixed for 5 min with 1% formaldehyde and permeabilised for 5 min in 0.1% Triton X-100 in PBS. Smears were sequentially incubated with the different antibodies. Secondary anti-rabbit and anti-mouse antibodies were conjugated with Alexa Fluor 488 and Alexa Fluor 594 (Molecular Probes, Invitrogen), respectively. Preparations were observed under a Nikon Elipse E1000 microscope with Metamorph image acquisition software.

Supporting Discussion.

A major difficulty of the RT-PCR and microarray analysis of invasion-related genes was that small differences in the proportion of parasites at different stages resulted in large differences in the abundance of transcripts of some genes that have a very sharp peak of expression, like members of the *pfRh* and *eba* families [16]. To overcome this difficulty, we developed an accurate procedure to obtain RNA preparations from parasites at precisely the same stage (see Materials and Methods).

To control for the amount of stage-specific cDNAs in RT-PCR experiments we used the single copy genes *rhoph2* and *ama1*, which have a similar timing of expression to *clag* genes or *eba* and *pfRh* genes, respectively. These controls revealed that the amount of cDNA of these stages was similar between the subclones (Fig. 4) and the variant levels of expression observed for several genes could not be explained by differences in the stage of the parasite populations from which the RNA was obtained, with the exception of the subclone 4D for which there was a lower concentration of

cDNA from genes expressed very late in the cycle like *ama1*. This can explain the lower abundance of transcripts of some members of the *eba* and *pfRh* families and of *acbp 10/15* in this subclone when compared with other subclones, but not the much lower abundance of *pfRh2b* transcripts. A transcript for *pfRh2b* could not be detected for the 4D subclone even when a 10 fold higher amount of template cDNA was used (data not shown). The gene *acbp-14*, which shows variant expression among subclones, also has a timing of expression similar to *ama1*, but other members of the *acbp* family have a timing of expression different to the controls used (*rhoph2* and *ama1*) [16]. With the procedure we used for the preparation of the RNAs it is unlikely that major differences exist in the amount of RNA of any stage between subclones, but differences in the expression of these genes among the subclones should be interpreted with caution (especially the lower expression of *acbp-10/15* in 4D, as discussed above).

Supporting References.

1. Crabb BS, Rug M, Gilberger TW, Thompson JK, Triglia T, et al. (2004) Transfection of the human malaria parasite *Plasmodium falciparum*. Methods Mol Biol 270: 263-276.
2. Cortes A, Benet A, Cooke BM, Barnwell JW, Reeder JC (2004) Ability of *Plasmodium falciparum* to invade Southeast Asian ovalocytes varies between parasite lines. Blood 104: 2961-2966.
3. Gentleman RC, Carey VJ, Bates DM, Bolstad B, Dettling M, et al. (2004) Bioconductor: open software development for computational biology and bioinformatics. Genome Biol 5: R80.
4. Gautier L, Cope L, Bolstad BM, Irizarry RA (2004) affy--analysis of Affymetrix GeneChip data at the probe level. Bioinformatics 20: 307-315.
5. Irizarry RA, Ooi SL, Wu Z, Boeke JD (2003) Use of mixture models in a microarray-based screening procedure for detecting differentially represented yeast mutants. Stat Appl Genet Mol Biol 2: Article1.
6. Smyth GK, Michaud J, Scott HS (2005) Use of within-array replicate spots for assessing differential expression in microarray experiments. Bioinformatics 21: 2067-2075.

7. Smyth GK, Speed T (2003) Normalization of cDNA microarray data. Methods 31: 265-273.
8. Crabb BS, Triglia T, Waterkeyn JG, Cowman AF (1997) Stable transgene expression in *Plasmodium falciparum*. Mol Biochem Parasitol 90: 131-144.
9. Mayer DC, Kaneko O, Hudson-Taylor DE, Reid ME, Miller LH (2001) Characterization of a *Plasmodium falciparum* erythrocyte-binding protein paralogous to EBA-175. Proc Natl Acad Sci USA 98: 5222-5227.
10. Lobo CA, Rodriguez M, Reid M, Lustigman S (2003) Glycophorin C is the receptor for the *Plasmodium falciparum* erythrocyte binding ligand PfEBP-2 (baebl). Blood 101: 4628-4631.
11. O'Donnell RA, Hackett F, Howell SA, Treeck M, Struck N, et al. (2006) Intramembrane proteolysis mediates shedding of a key adhesin during erythrocyte invasion by the malaria parasite. J Cell Biol 174: 1023-1033.
12. Holder AA, Freeman RR, Uni S, Aikawa M (1985) Isolation of a *Plasmodium falciparum* rhoptry protein. Mol Biochem Parasitol 14: 293-303.
13. Rayner JC, Galinski MR, Ingravallo P, Barnwell JW (2000) Two *Plasmodium falciparum* genes express merozoite proteins that are related to *Plasmodium vivax* and *Plasmodium yoelii* adhesive proteins involved in host cell selection and invasion. Proc Natl Acad Sci USA 97: 9648-9653.
14. Kaneko O, Lim BY, Iriko H, Ling IT, Otsuki H, et al. (2005) Apical expression of three RhopH1/Clag proteins as components of the *Plasmodium falciparum* RhopH complex. Mol Biochem Parasitol 143: 20-28.
15. O'Keeffe AH, Green JL, Grainger M, Holder AA (2005) A novel Sushi domain-containing protein of *Plasmodium falciparum*. Mol Biochem Parasitol 140: 61-68.
16. Bozdech Z, Llinas M, Pulliam BL, Wong ED, Zhu J, et al. (2003) The Transcriptome of the Intraerythrocytic Developmental Cycle of *Plasmodium falciparum*. PLoS Biol 1: 85-100.
