# Supplementary material for: Epigenetic Silencing of Plasmodium falciparum Genes Linked to Erythrocyte Invasion
Source: PLoS Pathog. 2007 Aug 3;3(8):e107. doi: 10.1371/journal.ppat.0030107 (PMC1937010; doi:10.1371/journal.ppat.0030107)
Supplement: Supplementary file 2 [file ppat.0030107.sg004.pdf]

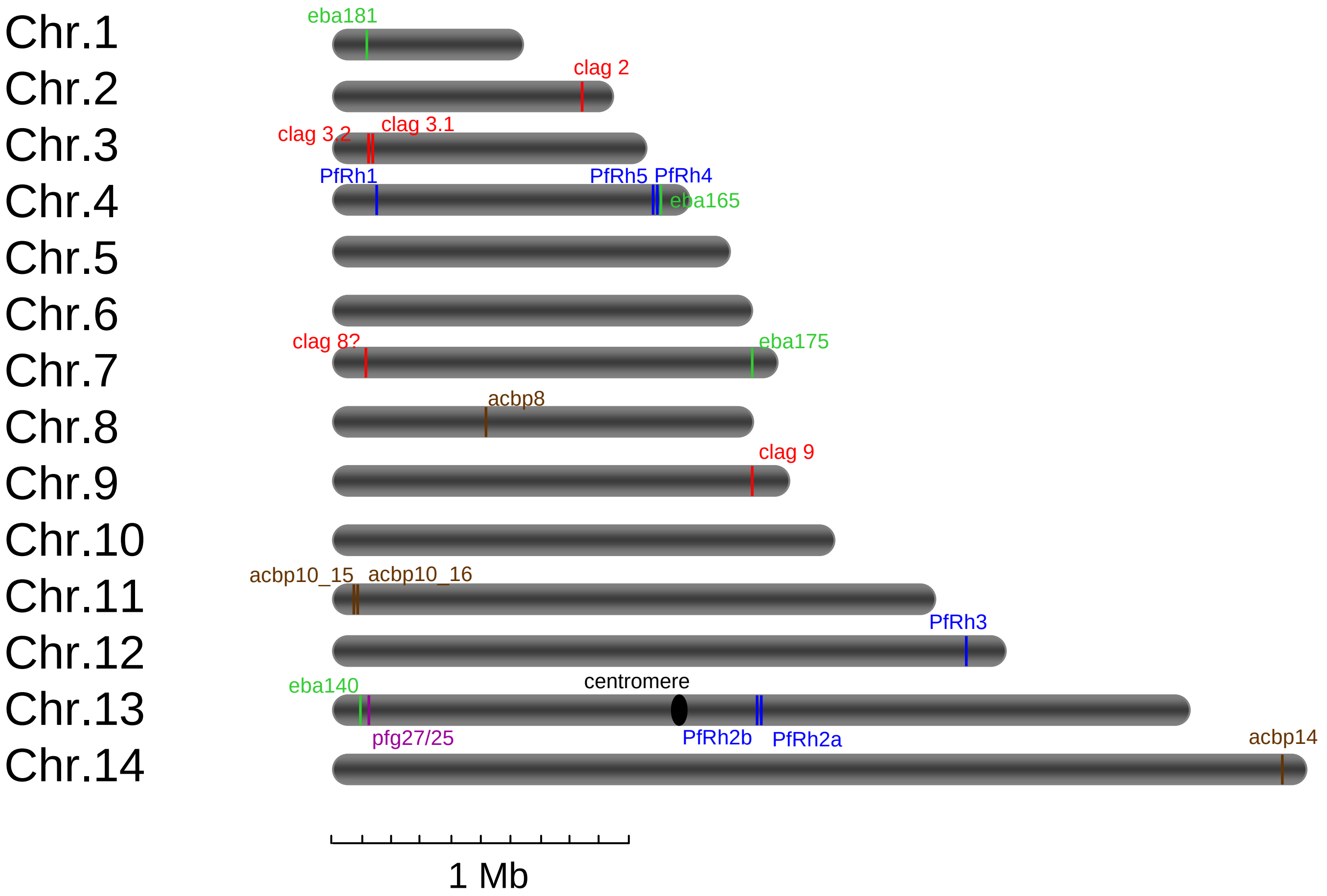
Chr.1
Chr.2
Chr.3
Chr.4
Chr.5
Chr.6
Chr.7
Chr.8
Chr.9
Chr.10
Chr.11
Chr.12
Chr.13
Chr.14
eba181
clag 2
clag 3.2
clag 3.1
PfRh1
PfRh5 PfRh4
eba165
clag 8?
eba175
acbp8
clag 9
acbp10_15
acbp10_16
PfRh3
eba140
centromere
pfg27/25
PfRh2b
PfRh2a
acbp14
1 Mb
